# Human organ donor-derived vagus nerve biopsies allow for well-preserved ultrastructure and high-resolution mapping of myelinated and unmyelinated fibers

**DOI:** 10.1038/s41598-021-03248-1

**Published:** 2021-12-13

**Authors:** Leif A. Havton, Natalia P. Biscola, Esther Stern, Plamen V. Mihaylov, Chandrashekhar A. Kubal, John M. Wo, Anita Gupta, Elizabeth Baronowsky, Matthew P. Ward, Deborah M. Jaffey, Terry L. Powley

**Affiliations:** 1 Departments of Neurology and Neuroscience, Icahn School of Medicine at Mount Sinai, New York, NY 10029 USA; 2 James J. Peters Department of Veterans Affairs Medical Center, Bronx, NY USA; 3 Department of Neurology, Icahn School of Medicine at Mount Sinai, New York, NY USA; 4 Department of Surgery, Indiana University School of Medicine, Indianapolis, IN USA; 5 Department of Medicine, Indiana University School of Medicine, Indianapolis, IN USA; 6 Department of Psychological Sciences, Purdue University, West Lafayette, IN USA; 7 Weldon School of Biomedical Engineering, Purdue University, West Lafayette, IN USA

**Keywords:** Myelin biology and repair, Peripheral nervous system

## Abstract

The vagus nerve provides motor, sensory, and autonomic innervation of multiple organs, and electrical vagus nerve stimulation (VNS) provides an adjunctive treatment option for e.g. medication-refractory epilepsy and treatment-resistant depression. The mechanisms of action for VNS are not known, and high-resolution anatomical mapping of the human vagus nerve is needed to better understand its functional organization. Electron microscopy (EM) is required for the detection of both myelinated and unmyelinated axons, but access to well-preserved human vagus nerves for ultrastructural studies is sparse. Intact human vagus nerve samples were procured intra-operatively from deceased organ donors, and tissues were immediately immersion fixed and processed for EM. Ultrastructural studies of cervical and sub-diaphragmatic vagus nerve segments showed excellent preservation of the lamellated wall of myelin sheaths, and the axolemma of myelinated and unmyelinated fibers were intact. Microtubules, neurofilaments, and mitochondria were readily identified in the axoplasm, and the ultrastructural integrity of Schwann cell nuclei, Remak bundles, and basal lamina was also well preserved. Digital segmentation of myelinated and unmyelinated axons allowed for determination of fiber size and myelination. We propose a novel source of human vagus nerve tissues for detailed ultrastructural studies and mapping to support efforts to refine neuromodulation strategies, including VNS.

## Introduction

The vagus nerve provides important motor, sensory, and autonomic innervation to multiple somatic, somatosensory, and visceral targets of the head, neck, thorax, abdomen, and pelvis. Vagus nerve stimulation (VNS) has emerged as a neuromodulation strategy to provide an adjunctive therapy for medication-refractory epilepsy^1,2^ and treatment-resistant depression^3,4^. Non-invasive approaches to electrically stimulate the vagus nerve have more recently emerged as an additional treatment strategy for migraine and cluster headache pain^5,6^. VNS may also inhibit cytokine production and modulate inflammatory conditions^7^. However, mechanisms of action for VNS-associated clinical effects across multiple medical conditions are not well understood.

In the peripheral nervous system (PNS), there is a close relationship between morphological features of individual nerve fibers and their physiological properties. For instance, information on the size and myelination of individual nerve fibers can be used to predict their conduction properties and assignment to different functional modalities and subsets of motor, sensory, and autonomic types^8–13^. Light and electron microscopy followed by data segmentation of individual nerve fibers in the PNS allows for the quantitative analysis of nerve fiber size and myelination as well as use of the morphological characteristics for the assignment of individual fibers to distinct functional groups^14^.

In order to improve on current electrode designs and refine stimulation strategies for optimized and expanded VNS applications to treat medical conditions, this rapidly expanding research field urgently needs a better understanding of the normal vagus nerve morphology, including the distribution and packaging of different myelinated and unmyelinated fiber types within individual nerve fascicles. Earlier studies across multiple species, including rodents, pigs, and humans, have shown extensive variability in the size and fascicular organization of the vagus nerve^15,16^. These species differences are of important consideration for the development of optimized electrical stimulation strategies, as thresholds for electrical activation and blocking of fibers in the vagus nerve are influenced by, for instance, the number and size of nerve fascicles as well as perineurium thickness and the areal distribution of the endoneurium^17^.

The vast majority of nerve fibers in the mammalian autonomic nervous system, including the human vagus nerve, are unmyelinated, requiring electron microscopy (EM) for their detection and morphological characterization^18–21^. However, ultrastructural studies on the number, size, and myelination of the human vagus nerve have been sparse. Limited access to well-preserved nervous tissues from younger adults represents a significant technical challenge for ultrastructural studies of the human peripheral nervous system, including the vagus nerve. To date, most morphological studies of the human vagus nerve have been performed using autopsy-derived tissues from elderly subjects and have been limited to light microscopic (LM) descriptions of nerve architecture and fascicular organization^15,22,23^. A recent study of human vagus nerve samples included abdominal vagus nerve samples from organ donors, but the analyses performed were also restricted to LM studies^16^.

We propose the use of human vagus nerve biopsies obtained from deceased organ donors as a novel source that allows for immediate tissue fixation and preservation of nervous tissues for detailed ultrastructural studies and mapping of the vagus nerve, including its myelinated and unmyelinated axons. This subject cohort includes healthy adults with a benign past medical history. The organ donors have recent brain death with preserved circulatory physiology to chest and abdominal organs unlike the lack of circulation and oxygenation of tissues in post-mortem studies. Ultrastructural data derived from studies of the vagus nerves from such organ donors will be in a unique position to guide the refinement of neuromodulation strategies using electrical stimulation, including VNS.

## Results

Human vagus nerve samples were collected intra-operatively from 27 consecutive organ donors following the diagnosis of brain death and procurement of organs for transplantation. Cervical, anterior and posterior abdominal, and gastric vagus nerve biopsies were obtained in male (n = 14) and female (n = 13) organ donors aged 44.3 ± 3.7 and 48.2 ± 3.1 years, respectively. There was no statistical difference in age between female and male donors. Immediate immersion fixation of the samples in a mixed aldehyde solution followed by tissue processing and embedding in a plastic resin allowed for LM studies of toluidine blue-stained sections from all subjects (n = 27). Additional EM studies of vagus nerve segments were performed on a subset of the subjects (n = 8).

**Figure 1:**
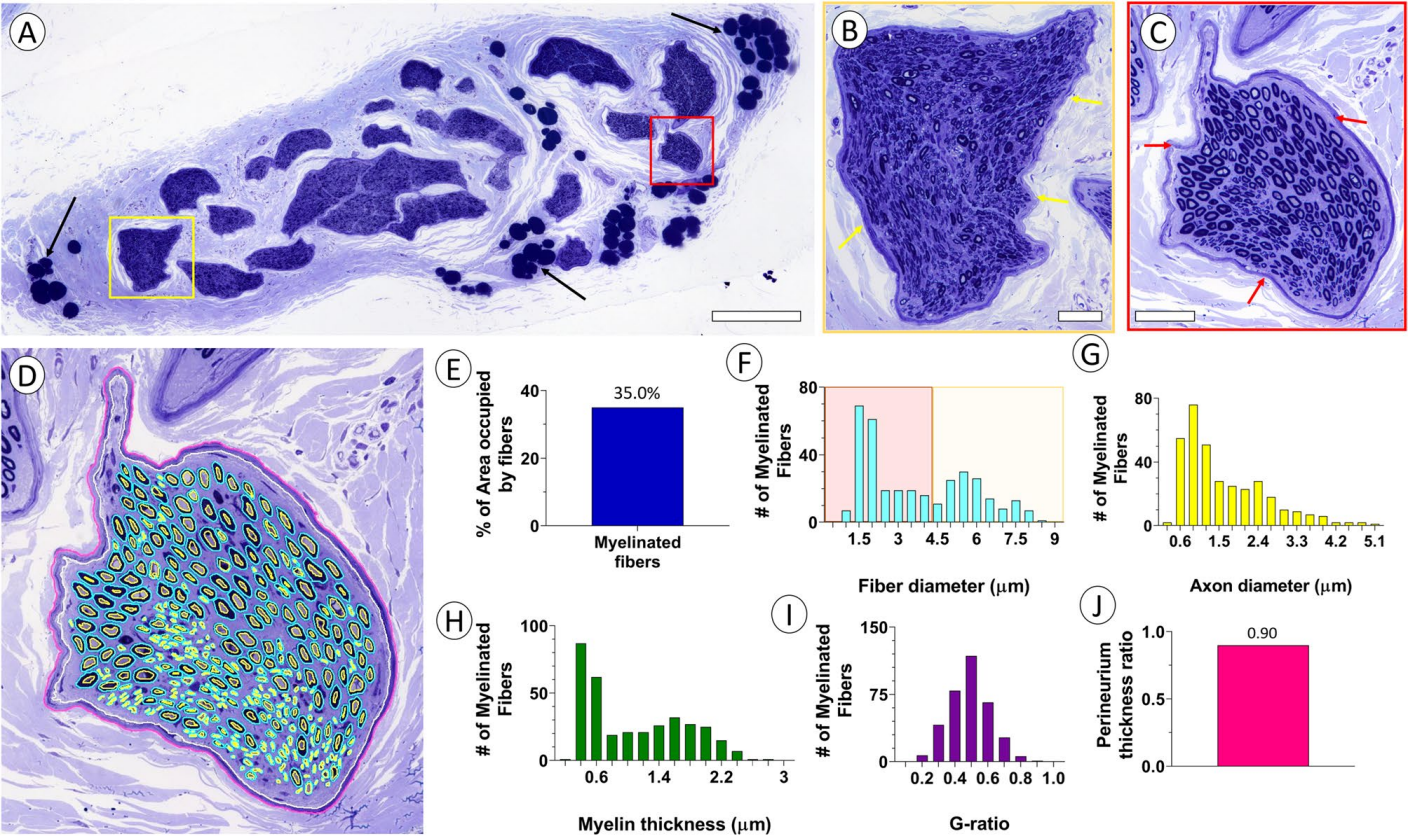
Light micrographs of human cervical vagus nerve. (**A**) LM overview of a representative cervical vagus nerve from a 49 year old female. Note multiple fascicles surrounded by loose connective tissue and lipid droplets (arrows). Scale bar = 500 µm. (**B**, **C**) Detailed view of individual fascicles indicated by yellow and red boxes in (**A**). Note distinct perineurium forming distinct boundary of each fascicle (arrows) and presence of numerous myelinated axons within each fascicle. Scale bars = 50 µm. (**D**) Fascicle in (**C**) after segmentation of the inner and outer contours of myelinated axons (n = 345) and perineurium. Quantitative information in (**E**–**J**) are derived from contoured structures in (**D**). (**E**) Histogram of percent of fascicle area inside of perineurium occupied by myelinated axons. (**F**–**H**) Size distribution of myelinated fiber diameter, axon diameter, and myelin thickness, respectively. Note two distribution peaks suggesting two distinct populations of myelinated fibers. (**I**) Distribution of G-ratios for myelinated fibers. (**J**) Histogram of perineurium thickness ratio.

LM of toluidine blue-stained sections of the distal cervical vagus nerve typically showed multiple fascicles, which varied extensively in size and included rounded, elongated, and irregular shapes (Fig. 1A–C). The fascicles were separated by a loose-textured epineurium and adipocytes. Each fascicle was outlined by a perineurium, which formed a distinct fibrous border. Numerous myelinated axons and intermittent Schwann cell nuclei populated each fascicle. Segmentation of the inner and outer myelin contours of myelinated axons and the perineurium allowed for quantitative studies of a representative cervical vagus nerve fascicle (Fig. 1D). The myelinated fibers (n = 345) occupied 35.0% of the endoneurium space in the transverse sectional plane (Fig. 1E). The myelinated fiber diameter and axon diameter were 3.43 ± 0.11 µm and 1.46 ± 0.05 µm, respectively. A size distribution display of the myelinated fiber and axon size showed two frequency peaks, one within the 0–4 µm and one within the > 4–9 µm myelinated fiber diameter range (Fig. 1F, G). The myelin thickness was 0.98 ± 0.03 µm with size distribution display demonstrating two peaks (Fig. 1H) and the G-ratio was 0.44 ± 0.01 (Fig. 1I). The perineurium thickness ratio, calculated as the endoneurium area divided by the area outlined by the outer surface of the perineurium was 0.90 (Fig. 1J).

**Figure 2:**
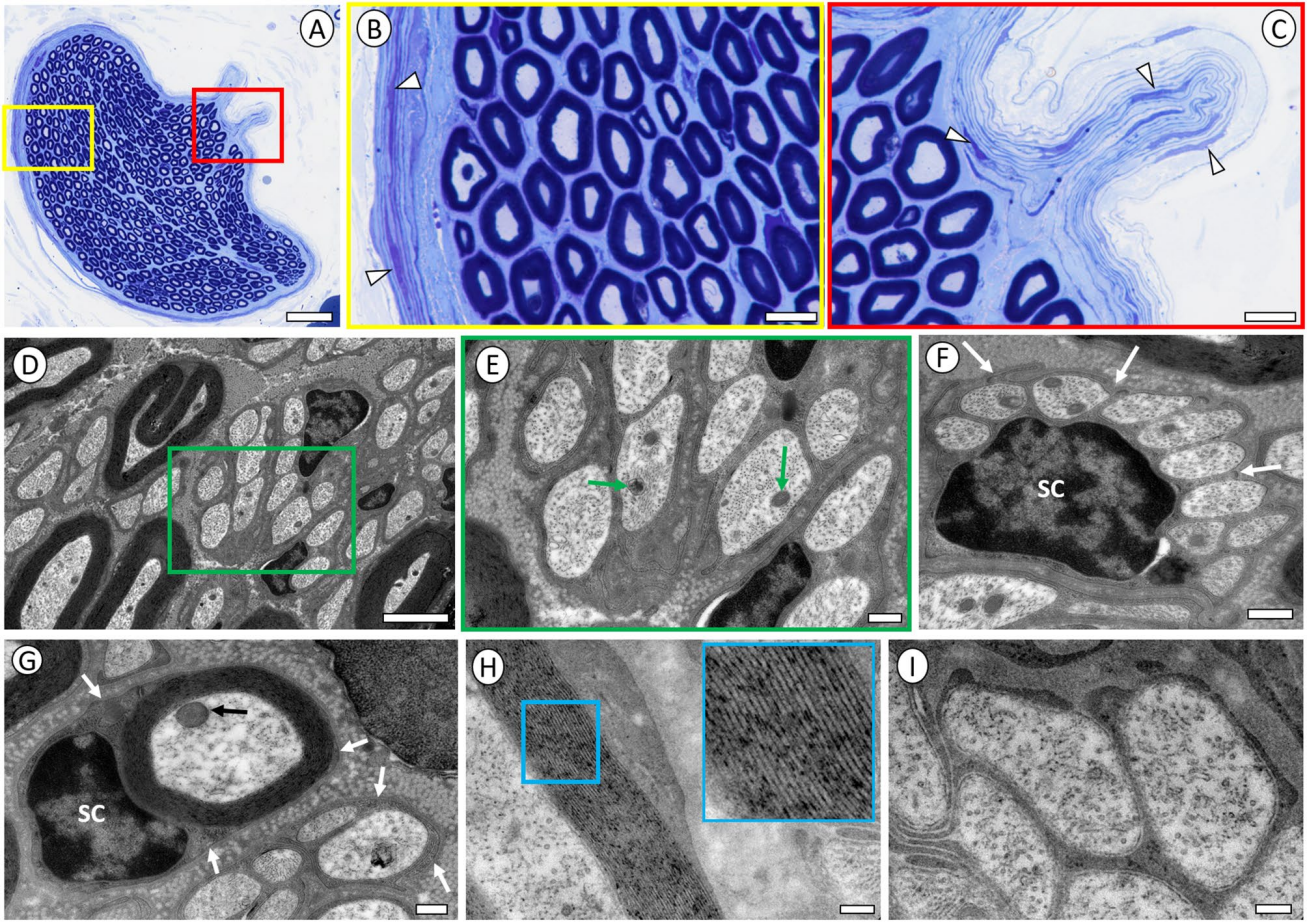
Light and electron micrographs of human cervical vagus nerve. (**A**) LM overview of representative fascicle from the cervical vagus nerve in 45 year old female. Scale bar = 50 µm. (**B**, **C**) Detailed view of yellow and red boxed areas in (**A**). Note lamellar organization of perineurial cells with epithelioid features forming multiple concentric layers and adjacent myelinated fibers. Small axonal bundles branching off the distal cervical vagus nerve may innerve thoracic target, including cardiac structures. Scale bar in B,C = 10 µm. Electron micrographs in (**D**–**G**) were obtained from same female subject as in (**A**–**C**). (**D**) Representative view of a mixed collection of myelinated and unmyelinated axons in the cervical vagus nerve. Scale bar = 2 µm. (**E**) Higher magnification of green box area in (**D**). Note well-preserved cytoskeleton, including microtubules and neurofilaments, and mitochondria (green arrows). Scale bar = 0.4 µm. (**F**) Remak bundle formed by Schwann cell (SC) and group of unmyelinated axons. Note basal lamina surrounding the Schwann cell plasma membrane (white arrows). Scale bar = 0.5 µm. (**G**) Schwann cell nucleus (SC) and associate myelinated axon. Note basal lamina surrounding the plasma membrane of myelinating Schwann cell and of adjacent Remak bundle. Scale bar = 0.4 µm. (**H**) Detail of myelin sheath with close-up view of area in blue box from cervical vagus nerve in a 49 year old male. Note integrity of myelin with lamellar wall structure and preservation of spacing between membrane layers. Scale bar = 0.2 µm. (**I**) Close-up view of unmyelinated axons in same subject as in (**H**) Note excellent preservation of microtubules, neurofilaments, plasma membrane, and surrounding basal lamina specialization. Scale bar = 0.2 µm.

The plastic-embedded and toluidine-blue stained sections allowed for the distinct LM visualization of well-preserved connective tissue, which forms the perineurium, and identification of some of its organizational features (Fig. 2A). Perineurial cells with epithelioid features formed multiple concentric layers with a lamellar organization and myelinated axons were readily encountered along the inner surface of the perineurium (Fig. 2B, C). EM studies of the cervical vagus nerve confirmed the presence of myelinated axons and demonstrated an additional large population of unmyelinated axons and Remak bundles interspersed between the myelinated fibers (Fig. 2D–G). The ultrastructure was well-preserved with demonstration of intact axonal membranes, cytoskeleton and organelles, including microtubules, neurofilaments and mitochondria. The myelin sheaths associated with myelinated fibers across a wide size range were also well preserved with an intact lamellar wall structure (Fig. 2H). All Schwann cell membranes were outlined by a basal lamina (Fig. 2I).

**Figure 3:**
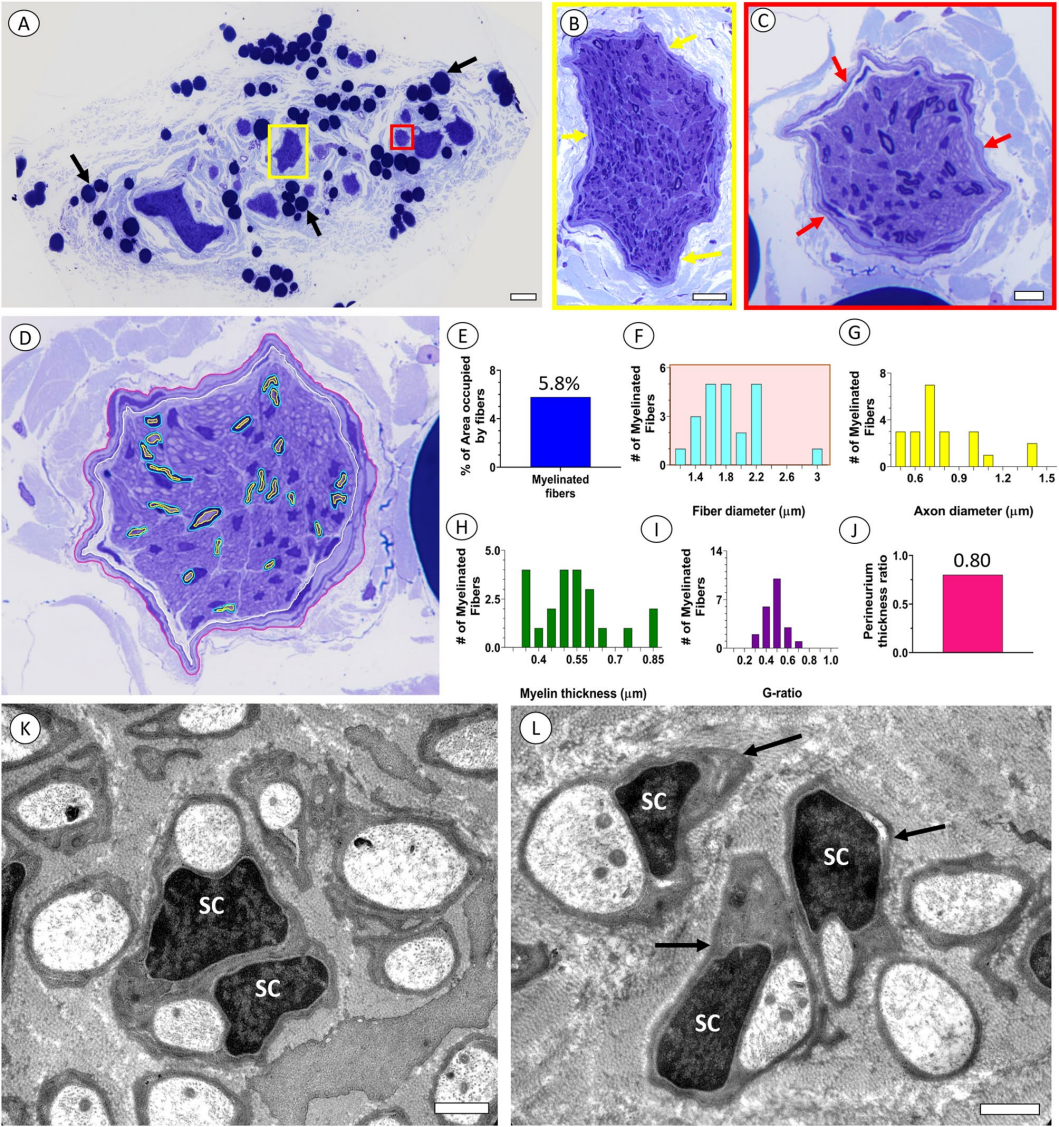
Light and electron micrographs of abdominal and gastric branches of human vagus nerve. (**A**) LM overview of a representative abdominal vagus nerve from a 49 year old female. Note multiple fascicles surrounded by loose connective tissues and lipid droplets (arrows). Scale bar = 100 µm. (**B**, **C**) Detailed view of individual fascicles indicated by yellow and red boxes in (**A**). Note outline of perineurium of each fascicle (arrows) and intermittent presence of myelinated axons within each fascicle. Scale bar in B = 25 µm; Scale bar in C = 10 µm. (**D**)**.** Fascicle in (**C**) after segmentation of the inner and outer contours of myelinated axons (n = 22) and perineurium. Quantitative information in (**E**–**J**) are derived from contoured structures in (**D**). (**E**) Histogram of percent of fascicle area inside of perineurium occupied by myelinated axons. (**F**–**H)** Size distribution of myelinated fiber diameter, axon diameter, and myelin thickness, respectively. (**I**) Distribution of G-ratios for myelinated fibers. (**J**) Histogram of perineurium thickness ratio. (**K**, **L**) Electron micrographs of unmyelinated axons of the abdominal vagus nerve in 52 year old male. (**K**) Note Remak bundle formed by Schwann cell with nucleus viewed as two portions (SC) in this plane of sectioning and two unmyelinated axons. (**L**) Schwann cell nuclei (SC) with associated unmyelinated axons surrounded by collagen fibers. Note basal lamina (arrows) outlining the cell membrane of each Schwann cell. Scale bar in K, L = 2 µm.

Toluidine blue-stained LM sections of both the anterior and posterior abdominal vagus nerve segments show readily identifiable fascicles, and each fascicle is enclosed by a perineurium (Fig. 3A–C). Within each fascicle, a modest number of myelinated axons of varied size and myelination was present. Most of the neuropil was devoid of myelinated fibers but showed an intermittent distribution of Schwann cell nuclei. Segmentation of the inner and outer myelin contours of myelinated axons and the perineurium allowed for quantitative studies of a representative abdominal vagus nerve fascicle (Fig. 3D). The myelinated fibers (n = 22) occupied 5.8% of the endoneurium space in the transverse plane (Fig. 3E). The myelinated fiber diameter and axon diameter were 1.75 ± 0.08 µm and 0.74 ± 0.05 µm, respectively, and a size distribution display showed all fibers within the 0–4 µm myelinated fiber diameter range (Fig. 3F, G). The myelin thickness was 0.50 ± 0.03 µm (Fig. 3H), and the G-ratio was 0.35 ± 0.04 (Fig. 3I). The perineurium thickness ratio was 0.80 (Fig. 3J). Ultrastructural studies of the abdominal and gastric vagus nerve segments confirmed the presence of a relatively sparse population of myelinated axons with excellent myelin sheath preservation and well-preserved axonal cytoskeleton, including neurofilaments and microtubules. A large number of unmyelinated fibers dominated the endoneurium space and, together with Schwann cells, formed Remak bundles (Fig. 3K, L). An abundance of collagen fibers was interspersed between the fibers and Schwann cells.

**Figure 4:**
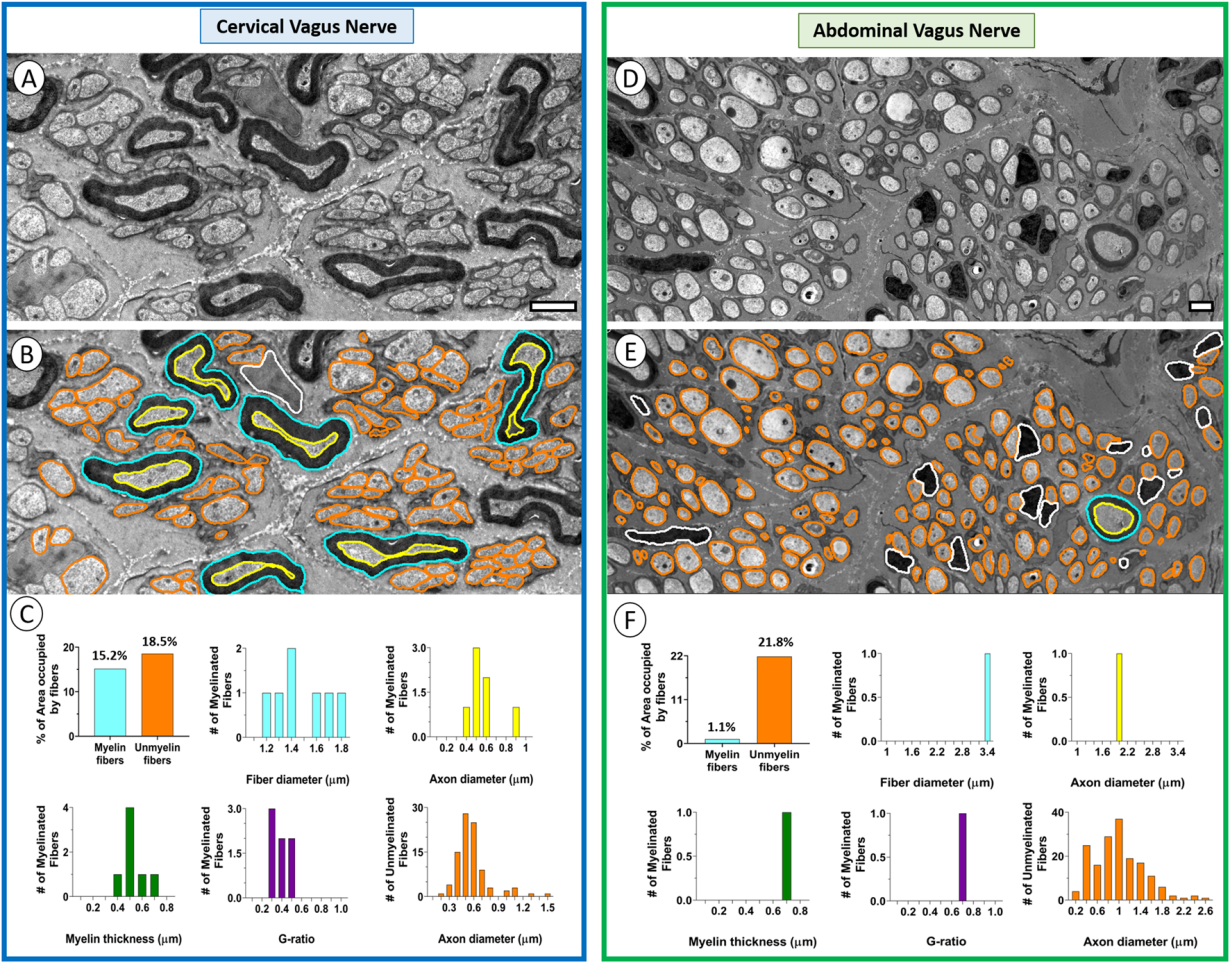
Data segmentation of myelinated and unmyelinated fibers of cervical and posterior abdominal segments of the human vagus nerve. (**A**) Electron micrograph of a representative area of the left cervical vagus nerve in a 49 year old male subject. Note small group of myelinated fibers surrounded by numerous unmyelinated axons and single Schwann cell nucleus. Scale bar = 2 µm. (**B**) The outer and inner circumferences of each myelin sheath was contoured individually and indicated in blue and yellow, respectively. All unmyelinated fiber outlines were contoured in orange. (**C**) Quantitative analysis derived from segmented data, including percent area occupied by myelinated and unmyelinated fibers, size distributions for fiber and axon proper diameters of myelinated fibers, myelin thickness, G-ratios for myelinated axons, and axon diameters for unmyelinated axons. (**D**) Electron micrograph of a representative area from the posterior abdominal branch of the vagus nerve in a 52 year old male subject. Note single myelinated fibers surrounded by numerous unmyelinated axons and Schwann cell nuclei. Scale bar = 2 µm. (**E**) The outer and inner circumferences of the single myelinated fiber were contoured and indicated in blue and yellow, respectively. All unmyelinated fiber outlines were contoured in orange. (**F**) Quantitative analysis derived from segmented data, including percent area occupied by myelinated and unmyelinated fibers, size distributions for fiber and axon proper diameters of myelinated fiber, myelin thickness, G-ratio for myelinated axon, and axon diameters for unmyelinated axons.

To show feasibility for using human organ-donor derived vagus nerve samples for quantitative analysis of fiber size and myelination, electron micrographs of representative areas were obtained from representative cervical and abdominal portions the vagus nerve and tiled, followed by computer-supported manual segmentation of myelinated and unmyelinated fibers and Schwann cell nuclei within the region of interest (Fig. 4A, B, D, E). For the cervical vagus nerve sample, the region of interest occupied by myelinated fibers (n = 7) and unmyelinated axons (n = 92) was 15.2% and 18.5%, respectively (Fig. 4C). In contrast, the region of interest for the abdominal vagus nerve occupied by myelinated fibers (n = 1) and unmyelinated axons (n = 170) was 1.1% and 21.8%, respectively (Fig. 4F). For the cervical vagus nerve, the myelinated fiber and axon diameters were 1.43 ± 0.08 µm and 0.50 ± 0.06 µm, respectively (Fig. 4C). The myelin thickness and G-ratio were 0.47 ± 0.04 µm and 0.35 ± 0.04, respectively. The unmyelinated fiber diameter was 0.52 ± 0.02 (Fig. 4C). For the abdominal vagus nerve, the single myelinated fiber and axon diameters were 3.20 µm and 1.96 µm, respectively Fig. 4F). The myelin thickness was 0.62 µm and the G-ratio was 0.61 (Fig. 4F). The unmyelinated fiber diameter was 0.89 ± 0.03 µm (Fig. 4F).

**Figure 5:**
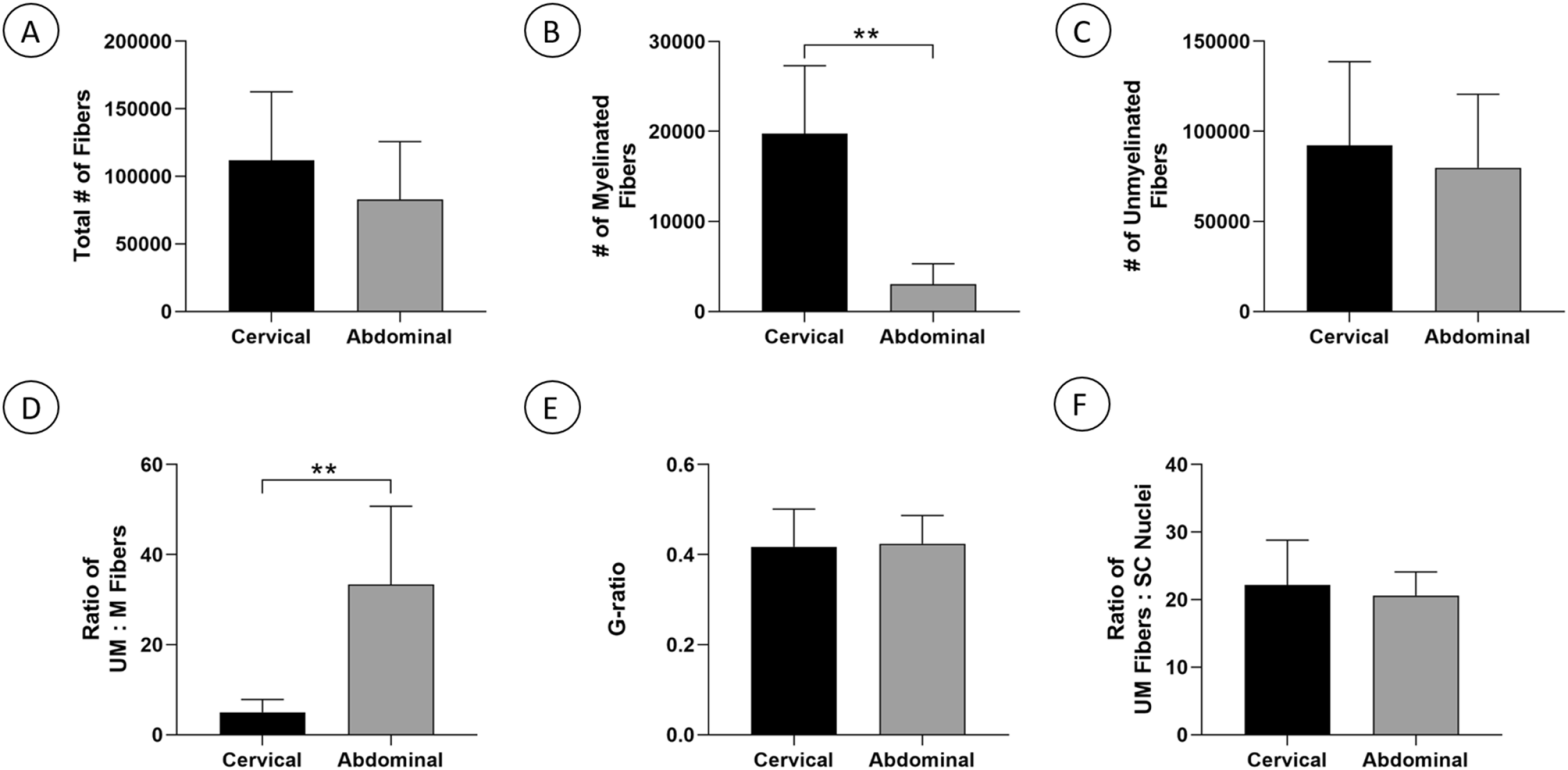
Quantitative transmission electron microscopy data on myelinated and unmyelinated fibers of cervical and abdominal segments of the human vagus nerve. (**A**) Total number of myelinated and unmyelinated fibers. (**B**) Number of myelinated fibers. Please note a significantly higher number of myelinated fibers in the cervical compared to abdominal vagus nerve (*p* = 0.0025). (**C**) Number of unmyelinated fibers. (**D**) Ratio of unmyelinated (UM) to myelinated (M) fibers. Please note a significantly higher number of UM to M fibers in the abdominal compared to cervical vagus nerve (*p* = 0.0025). (**E**) G-ratio calculated ratio of axonal diameter/myelinated fiber diameter. (**F**) Ratio of UM fibers to Schwann cell nuclei.

The total number of nerve fibers in cervical and abdominal branches of the human vagus nerve was calculated by extrapolating the number of segmented myelinated and unmyelinated axons in a region of interest (ROI) from a representative fascicle at each nerve biopsy site. There was extensive variability in the number of fibers at both the cervical and abdominal sites (Table 1). The total number of nerve fibers in the cervical vagus was 111,984 ± 19,217 (n = 7) and not significantly different from the corresponding total number nerve fibers of 82,861 ± 19,217 in the abdominal vagus (n = 5; Fig. 5A). The number of myelinated fibers in the cervical vagus nerve was 19,990 ± 2851 (n = 7) and significantly higher than the number of myelinated fibers of 3000 ± 1209 in the abdominal vagus nerve (n = 5; p = 0.0025; Fig. 5B). The number of unmyelinated fibers was 92,214 ± 17,589 for the cervical vagus nerve (n = 7) and was not different from the number of unmyelinated fibers of 79,861 ± 18,210 for the abdominal vagus nerve (n = 5; Fig. 5C). The ratio of unmyelinated to myelinated fibers was 5.0 ± 1.1 for the cervical vagus nerve (n = 7) and significantly higher than the corresponding ratio of 33.4 ± 7.8 for the abdominal vagus nerve (n = 5; p = 0.0025; Fig. 5D). The G-ratio for myelinated fibers was 0.41 ± 0.08 for the cervical vagus nerve and not different from the corresponding G-ratio of 0.42 ± 0.06 for the abdominal vagus nerve (n = 5; Fig. 5E). In an assessment of Remak bundle size, the ratio of unmyelinated axons to Schwann cell nuclei without an association with a myelinated axon was 22.2 ± 2.5 for the cervical vagus nerve (n = 7) and not significantly different from the corresponding ratio of 20.6 ± 1.6 for the abdominal nerve (n = 5; Fig. 5F).

**Table 1:** Summary of transmission electron microscopy studies of segmented myelinated and unmyelinated axons of the cervical and abdominal human vagus nerve.

Subject	Sex	Age (years)	Vagus nerve site	Total number of nerve fibers	Number of myelinated fibers	Number of unmyelinated fibers	Ratio of UM:M fibers	Ratio of UM fibers:Schwann cell nuclei
**Human cervical vagus nerve**								
1	Male	47	Left Cervical	42,285	13,405 (31.7%)	28,880 (68.3%)	2.2	17.8
2	Male	49	Left Cervical	95,386	8850 (9.3%)	86,536 (90.7%)	9.8	32.4
3	Female	47	Right Cervical	173,184	20,832 (12.0%)	152,352 (88.0%)	7.3	19.2
4	Female	50	Right Cervical	125,726	31,320 (24.9%)	94,406 (75.1%)	3.0	13.7
5	Female	49	Left Cervical	52,410	16,499 (31.5%)	35,910 (68.5%)	2.2	18.5
6	Female	49	Right Cervical	136,533	22,172 (16.2%)	114,361 (83.8%)	5.2	25.3
7	Female	45	Right Cervical	158,361	25,309 (16.0%)	133,052 (84.0%)	5.3	28.3
Mean ± SE		48.0 ± 0.6		111,984 ± 19,130	19,770 ± 2851 (20.2%)	92,214 ± 17,589 (79.8%)	5.0 ± 1.1	22.2 ± 2.5
**Human abdominal vagus nerve**								
1	Male	45	Anterior abdominal	45,064	1676 (3.7%)	43,388 (96.3%)	25.9	17.7
2	Male	47	Anterior abdominal	52,554	806 (1.5%)	51,747 (98.5%)	64.2	19.4
3	Male	52	Posterior abdominal	81,550	2739 (3.4%)	78,811 (96.6%)	28.8	21.3
4	Female	37	Anterior abdominal	81,324	2967 (3.6%)	78,356 (96.4%)	26.4	26.4
5	Female	63	Posterior abdominal	153,814	6813 (4.4%)	147,001 (95.6%)	21.6	18.3
Mean ± SE		48.8 ± 4.3		82,861 ± 19,217	3000 ± 1209 (3.3%)	79,861 ± 18,210 (96.7%)	33.4 ± 7.8	20.6 ± 1.6

*UM* unmyelinated fibers, *M* myelinated fibers.

## Discussion

EM studies of the human vagus nerve are associated with technical challenges and limitations. Prior ultrastructural investigations of autopsy-derived human vagus nerves have identified an early post-mortem loss of nerve fiber integrity and that unmyelinated fibers are particularly vulnerable to pre-fixation autolysis^24^. The post-mortem compromise of unmyelinated fiber integrity represents a particular challenge to studies of the human vagus nerve with its predominantly unmyelinated nerve fiber population, as unmyelinated fibers in both animal models and humans represent approximately 85% of all fibers in the cervical portion of the vagus nerve and typically more than 99% of all fibers in sub-diaphragmatic portions of the vagus nerve, including its abdominal branches^18–20,25^. The present study proposes a new approach with intra-operative harvesting of vagus nerve biopsies in organ donors and immediate tissue fixation and processing to allow for excellent preservation of the biopsy material and detailed ultrastructural studies of both myelinated and unmyelinated fibers (Fig. 5). In our experience, the fine structure encountered in the human organ donor-derived vagus nerve tissue is, from a tissue quality and preservation perspective, comparable to the ultrastructure demonstrated in studies of the peripheral and central nervous system following perfusion or immersion fixation across multiple species, including mice, rats, cats, pigs, and non-human primates^14,26–32^.

Solid organ transplantation has provided a path to increased survival and quality of life for patients with end-stage organ failure, including the heart, liver, lungs, and kidneys^33^. The acceptance of the concept of brain death as a medical and legal definition of a deceased patient has allowed for an increased procurement and transplantation of physiologically well perfused organs^34^.The procurement of solid organs in the chest and abdominal cavities of organ donors also provides access to several peripheral nervous structures, including the vagus nerve and its thoracic and abdominal branches. The intra-operative collection of vagus nerve biopsies after completed procurement of transplant organs allows for detailed morphological studies of the human peripheral nervous system. Several unique demographic, health, and tissue quality features are associated with the collection of such peripheral nerve samples. For instance, potential solid organ donors undergo careful clinical evaluation before organ harvesting to ensure optimal organ perfusion and laboratory testing for multiple viral agents to prevent spread of infectious disease according to U.S. federal guidelines provided by the Centers for Disease Control and Prevention (CDC) and the Organ Procurement and Transplantation Network (OPTN).

The underlying cause of brain death in organ donors is commonly an accident or a self-inflicted injury in a relatively young subject without chronic or progressive medical conditions. The demographic and medical history of the present subjects support this notion with an average age in the mid-forties and absence of any known neurodegenerative conditions, chronic infectious disease, or malignancy. In addition, the intra-operative harvesting of vagus nerve biopsies, immediate immersion fixation of the samples in a mixed aldehyde solution, and subsequent processing and plastic embedding of the nervous tissues allows for excellent tissue preservation before tissue sectioning and morphological analysis, including detailed ultrastructural examination of myelinated and unmyelinated nerve fibers.

Autopsy-derived nervous tissues represent an alternative source of peripheral nerves, including the vagus nerve, for morphological studies. Such samples may also provide important LM information about the organization and structure of the peripheral nervous system. However, important features with regards to demographics and past medical history need to be considered when interpreting autopsy-derived peripheral nerve specimens, as autopsy-derived tissues are generally obtained after an in-hospital patient death. Most patients are hospitalized due to serious acute and/or chronic medical conditions, and hospital-based deaths may result from irreversible disease progression despite therapeutic interventions or medical complications. The average age of expired patients undergoing a post-mortem examination is expected to be higher than the age of organ donors with a brain death diagnosis. Recent morphological studies of autopsy-derived samples of vagus nerve tissues highlight this notion, as the average age at death in these cohorts was in the mid- to upper-eighties, and a large portion of the subjects had a past medical history of malignancy or significant metabolic conditions^15,22,23^. Maintained oxygenated circulation to peripheral nervous tissue immediately prior to its procurement in the organ donors also provides a significant advantage for the preservation of ultrastructure in peripheral nerves compared to the delayed collection of similar tissues during an autopsy procedure.

Sampling of nerve biopsies from an elderly cohort raises several potential concerns, as numerous pre-existing medical conditions with known negative effects on the health of peripheral nerves are associated with advancing age. For instance, metabolic syndrome and diabetes mellitus are common with aging and known risk factors for peripheral neuropathy^35–37^. The presentation of a diagnosis of cancer is also more frequent in the elderly. Although an individual malignancy may contribute to a neuropathy, multiple treatment modalities for malignancies may more commonly compromise peripheral nerve health. For instance, radiation therapy applied to the neck and thorax may induce peripheral neuropathy, including mononeuropathy of the vagus nerve^38–40^. Drug-induced neurotoxicity, including a painful peripheral neuropathy, is a common adverse effect during the treatment course of many malignancies and may require dose reduction or cessation of chemotherapeutic agents^41,42^. Aging in the absence of a significant metabolic, cancer, or toxic exposure history may be an independent risk factor for neuropathic changes in the nervous system, as a marked portion of the aging population show signs of an idiopathic peripheral neuropathy^43^. Sonographic evaluations of the vagus nerve have also reported a decreasing cross-sectional area of the vagus nerve with increasing age^44^. Sampling of vagus nerve biopsies from a younger cohort of organ donors markedly reduces this risk of decreased peripheral nerve health from aging, pre-existing conditions, and neuro-toxic exposures from prior medical treatments.

Previous studies have made estimates on the number and size distribution of vagus nerve fibers and myelination in human samples using an LM approach and concluded that the proportion of myelinated fibers is much higher in the cervical vagus nerve compared to abdominal vagus nerve branches^16,45,46^. The present EM study provides additional support for the notion that myelinated fibers are more numerous at the cervical compared to abdominal levels also for the human vagus nerve, a finding that is also in agreement with earlier ultrastructural studies of the vagus nerve in cats^47^. However, for both the cervical and abdominal branches of the human vagus nerve, the vast majority of axons are unmyelinated. Based on quantitative EM studies in the cat, it is estimated that over 80% of the about 55,000 axons in the cervical vagus nerve are unmyelinated^47^. Similarly, we calculated that about 80% of the over 110,000 axons in the human cervical vagus nerve are unmyelinated. The organization of unmyelinated axons in Remak bundles may also differ along the course of the vagus nerve. The number of unmyelinated axons per Schwann cell was significantly higher in the supranodose compared to the infranodose portion of the cervical vagus nerve, whereas no difference in this ratio was detected between the infranodose and diaphragmatic segments of the vagus nerve in cats^47^. The present study provides support to the earlier study, as we report no significant difference in the number of unmyelinated axons per Schwann cell nucleus between the distal cervical and abdominal segments of the human vagus nerve.

Both the cervical and abdominal human vagus nerve samples showed marked inter-subject variability as well as extensive overlap between males and females with regards to the number of myelinated and unmyelinated fibers. The ultrastructural data from male and female organ donors were therefore pooled. Both biological and methodological aspects may have contributed to the observed extensive variability of nerve fiber numbers and myelination. First, ultrastructural studies were performed using an ROI within a single fascicle per site and subject. Here, it is possible that different fascicles may exhibit varied relative compositions of myelinated and unmyelinated fibers based on each peripheral target organ of innervation. Second, myelinated and unmyelinated fibers may not be evenly distributed across the cross section of individual fascicles. Third, there may be unrecognized organizational differences for myelinated and unmyelinated axons at the ultrastructural level between males and females. The present study design was not powered for the evaluation of possible sexual dimorphism, and future studies with a markedly larger number of EM samples will be needed to address this important aspect of the functional organization of the human vagus nerve.

Marked inter-subject variability was also shown in the present studies for the number of myelinated and unmyelinated axons in samples from left and right vagus nerve segments and between anterior and posterior abdominal branches. Earlier studies of the human vagus nerve did not detect differences between sides at the cervical level based on light microscopic analysis of overall nerve size or number of fascicles^15,22,23,48,49^. To address the possibility of vagus nerve laterality with regards to nerve fiber numbers at the cervical or sub-diaphragmatic levels in human samples, an expanded sample size will be needed for future ultrastructural studies.

We conclude that vagal nerve biopsies from organ donors represent a new source of nervous tissues for combined LM and EM studies that allows for excellent ultrastructure and detailed analysis of both myelinated and unmyelinated nerve fibers. Organ donors also represent a source of tissues from younger subjects and with fewer comorbidities compared to tissues historically obtained from autopsies in a hospital setting. Organ donor-derived vagus nerve tissues are in a unique position to contribute to an improved understanding of the morphological and functional organization of the vagus nerve and thereby contribute to the refinement of vagus nerve neuromodulation in clinical studies and use.

## Methods

**Figure 6:**
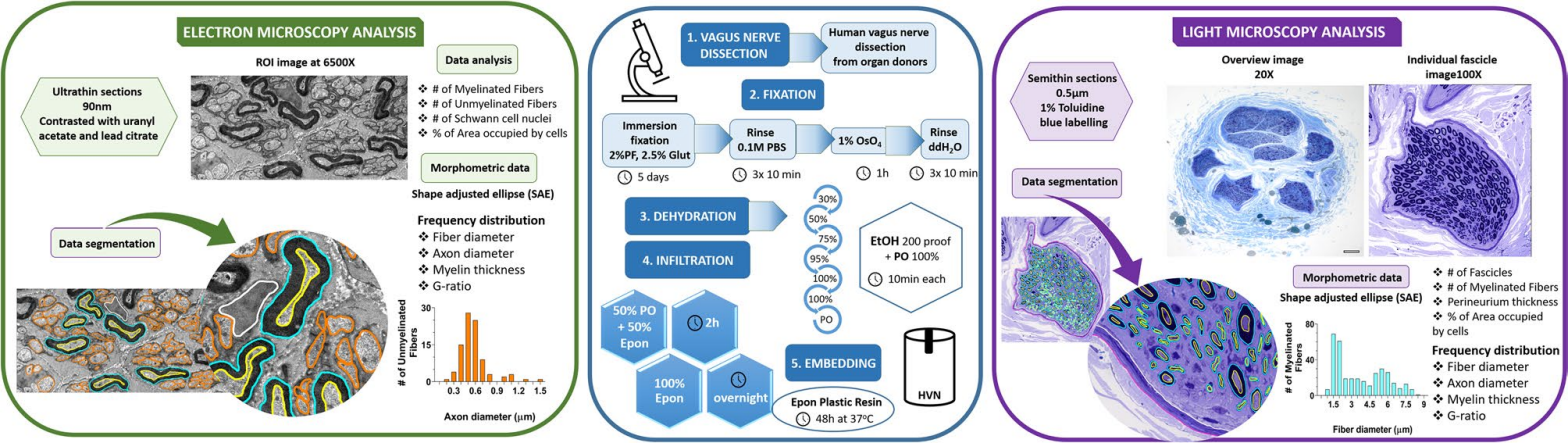
Summary of tissue processing steps and analysis of human organ donor-derived vagus nerve samples for LM and EM studies. Please note that the tissue fixation and embedding steps of human vagus nerve tissues are shared. The tissue sectioning of embedded tissue blocks, contrasting or staining, and imaging are performed according to LM- and EM-specific protocols. Data segmentation for morphological mapping studies and quantitative analysis of fiber at both LM and EM levels are performed using same approaches, including SAE corrections of axonal size and myelination. *HVN* human vagus nerve, *ROI* region of interest, *PO* propylene oxide, *PBS* phosphate buffered saline, *PF* paraformaldehyde, *Glut* glutaraldehyde.

Human vagus nerve samples were collected and processed for a combination of LM and EM studies for detailed analysis and mapping of myelinated and unmyelinated fibers (Fig. 6). For this purpose, vagus nerve biopsies were obtained intra-operatively from organ donors (n = 27). Separate consents for organ donation and research were obtained. Informed consent for study participation was obtained from a parent and/or legal guardian. No organ donors were prisoners. A diagnosis of brain death had been previously established by independent medical professionals according to State of Indiana and institutional regulations. All procedures related to tissue procurement and research use for tissues were in compliance with and approved by regulatory oversight review committees at University of Indiana School of Medicine, Indiana Donor Network, and Purdue University. The vagus nerve biopsies were obtained from a continuous series of qualified organ donors meeting the diagnostic and medical criteria for organ donation and with consent for research participation in place. Donors in whom lungs and stomach containing multivisceral transplants were pursued were excluded from the research studies. The research team was not involved with the identification and selection of organ donors.

After the completion of transplant organ harvesting, biopsies of the vagus nerve were collected. Approximately 10–20 mm long segments of the left and right cervical vagus nerve, anterior and posterior abdominal branches of the vagus nerve, and gastric vagus nerve branch were harvested. All vagus nerve segments were immediately placed in a fixative solution containing 2% paraformaldehyde and 2.5% glutaraldehyde for 5 days at 4 °C with daily changes of fixative solutions and with specimens kept on an oscillating plate during the immersion fixation process. The tissues were next rinsed in phosphate buffer at 4 °C overnight, osmicated in 1% osmium tetroxide, rinsed in H2O, dehydrated in 30%, 50%, 75%, 95%, and 100% ethanol, infiltrated in 50% propylene oxide (PO) + 50% Epon, and embedded in 100% Epon.

Plastic resin-embedded vagus nerve tissues were sectioned in the transverse plane at 0.5 µm thickness, stained with a 1% toluidine blue solution, mounted onto glass slides, and cover-slipped for light microscopy (LM). A Nikon E600 light microscope equipped with a DS-Fi3 camera (Nikon) was used for image capturing at 100X and tiling for the generation of photo-montages. The LM montages provided initial assessments of tissue integrity, technical quality of sections, and identification of regions of interest for the selection of subsequent ultrastructural studies.

Ultrathin sections at 70–90 nm in the transverse plane were obtained from Epon-embedded vagus nerve tissue blocks using an RMC PowerTome Ultramicrotome (Boeckeler Instruments^®^) equipped with an Ultra 45° 4.0 mm diamond knife (Diatome US^®^). The ultrathin sections were collected on formvar-coated single slot copper grids, contrasted with uranyl acetate and lead citrate, and analyzed in a transmission electron microscope (TEM) operating at 80 kV (Tecnai G2 Spirit Twin, FEI^®^, ThermoFisher Scientific^®^). Electron micrographs of regions of interest (ROI) were collected at 6500–42,000 × magnification using a Gatan Orius SC 1000B digital camera (Gatan^®^, Inc.), and tiling of composite EM images into montages was performed using Adobe Photoshop^®^ (version: 21.1.3 20200508) or Image Composite Editor (ICE^®^, Microsoft).

Data segmentation of individual myelinated and unmyelinated axons was performed manually or semi-automatically using Neurolucida^®^ 360 (MBF Bioscience). For myelinated fibers, both the outer and inner contours of each myelin sheath within the ROI were segmented to allow for the calculation of both fiber and axon diameters, whereas segmentation of corresponding unmyelinated axons included only the axon proper outline for each fiber. Raw data files for the cross-sectional area, perimeter, and centroid were next obtained for each segmented nerve fiber enclosure. Diameters for myelinated and unmyelinated axons were calculated based on the shape-adjusted ellipse (SAE) correction approach, using cross-sectional area and perimeter measurement data to calculate the minor diameter of an ellipse^14^. The G-ratio is the ratio of the SAE-calculated diameters of the axon proper and the corresponding myelinated fiber using the centroids for both structures to facilitate the paired linkage^14^. The segmented data were used for ultrastructural analysis and SAE-corrected calculations of fiber and axon diameters, G-ratio, and myelin thickness as (fiber diameter − axon diameter)/2. The perineurium thickness ratio was calculated as the endoneurial area divided by the area outlined by the outer surface of the perineurium.

To provide estimates of the total number of fibers in the human vagus nerve as well as contributions by myelinated and unmyelinated axons, a representative fascicle was selected at multiple cervical and abdominal locations. A rectangular region of interest (ROI) was next placed in the mid portion of each fascicle, guided by the centroid location for each fascicle. The ROI was 5435 ± 234 µm^2^ (n = 7) for the cervical and 4661 ± 862 µm^2^ (n = 5) for the abdominal samples. All myelinated and unmyelinated axons as well as Schwann cell nuclei were manually contoured and quantified within all ROIs. Next, the total endoneurium area was determined for each selected cervical and abdominal nerve segment as the summed areas determined by the inner perineurium outlines for all fascicles at each biopsy site. The total number of myelinated and unmyelinated axons was subsequently calculated as an extrapolation based on the quantitative data provided by each ROI and cross sectional areas. To provide an estimate of Remak bundle size the total number of unmyelinated axons was divided by the number of Schwann cell nuclei not associated with a myelinated axon for each ROI.

All data are presented as mean ± standard error (SE). The non-parametric ANOVA One-way testing followed by the Kruskal–Wallis test and Dunn’s multiple comparison test were performed for comparisons between groups using Prism8^®^ (GraphPad Software, Inc, La Jolla, CA). A value of *p* < 0.05 was considered to reflect a statistically significant difference between groups. Frequency distributions for all morphometric size analyses were calculated and plotted using Prism8^®^.
